# Assessing the efficacy of cinnamon compounds against *H*. *pylori* through molecular docking, MD Simulations and ADMET analyses

**DOI:** 10.1371/journal.pone.0299378

**Published:** 2024-03-11

**Authors:** Muhammad Farhan Sarwar, Afnan Zahra, Mudassar Fareed Awan, Sajed Ali, Muhammad Shafiq, Khursheed Muzammil

**Affiliations:** 1 Department of Biotechnology, Knowledge Unit of Science (KUSC), University of Management and Technology Sialkot, Sialkot, Pakistan; 2 Department of Chemistry, Government College for Women University Sialkot (GCWUS), Sialkot, Pakistan; 3 Department of Public Health, College of Applied Medical Sciences, Khamis Mushait Campus, King Khalid University, Abha, Kingdom of Saudi Arabia (KSA); Universidad Autonoma de Chihuahua, MEXICO

## Abstract

Antibiotics are the drugs that are used for the management of microbial diseases. However, these conventional synthetic drugs can harmfully affect the human health. Since phytochemicals are extracted from natural sources and, are hence relatively safer for human health, they are the enticing alternatives in this regard. Cinnamon is also one of those plants which is being employed as herbal medication for centuries against certain microbial infections due its significant therapeutic effectiveness. A well-known pathogenic bacterium called *H*. *pylori* causes a wide range of illnesses in human body. This pathogen’s pathogenicity is determined by certain virulent proteins. In this study, some of such proteins, which included *virB4*, *virB8*, and *virB9* were selected to evaluate the therapeutic efficiency of cinnamon compounds. These proteins were identified in different isolates of *H*. *pylori*. The structural modelling of all these proteins were performed initially in order to proceed them for molecular docking analysis. While, the docking studies illustrated that one of the cinnamon compounds, *cinnamyl acetate*, showed significant binding interactions with *virB4* and *virB9*. However, *benzyl benzoate* which is another cinnamon compound, docked well with *virB8*. Afterwards, the MD simulations were incorporated to explore the interaction motions and structural stability of all the docked complexes. In this regard, the resultant maps of Bfactor, eigenvalues and elastic network model, among other factors ensured the structural stabilities of all the respective complexes. After these crucial estimations, *benzyl benzoate* and *cinnamyl acetate* underwent the ADMET investigation to assess their pharmacokinetic characteristics. SwissADME and ADMETLab 2.0 server were employed for this investigation. The compiled findings these servers revealed that both, *benzyl benzoate* and *cinnamyl acetate*, exhibited a significant level of pharmacokinetic and drug-likeness conformity.

## Introduction

*Helicobacter pylori* is a gram-negative, microaerophilic spiral bacterium which is commonly found in the stomach [1]. It has been associated with stomach cancer, esophageal cancer, colon cancer, rectum cancer, and ophthalmic cancer, as well as infections of lymphoid tissue in the stomach [2, 3]. It has been reported that 90% of the patients do not exhibit or experience any sort of complications and other severe indications of the disease [4]. But, the individuals with *H*. *pylori* mediated infections have 10% to 20% of the probability of developing lifelong peptic ulcers [5, 6]. According to a reported study, different virulent factors have been reported in this pathogen which impart virulence to *H*. *pylori* or assist it in developing the virulence. These proteins are primarily the secretion system proteins which include *virB4*, *virB8* and *virB9* and they have been reported to be found in different strains of *H*. *pylori* [7].

Whereas, the drugs to treat *H*. *pylori* infections are concerned, clarithromycin, metronidazole, and amoxicillin are the most significant antibiotics in this context [8]. But, it has also been illustrated that microorganisms develop resistance against antibiotics due to their extensive use [9] including *H*. *pylori*, which has also shown antibiotic resistance against specific antibiotics [10, 11]. Therefore, due to this continuously increasing antibiotic resistance, there is a need to adopt unconventional and advanced therapeutic approaches to treat *H*. *pylori* infections [12]. Such strategies may involve certain plant extracts or other portent compounds which also possess antimicrobial or anti-bacterial properties [13]. The anti-bacterial characteristic is actually imparted by the secondary metabolites and certain flavonoids [14, 15] which are present in different proportions in such plants. The advantage of these phytochemicals was evaluated in terms of being safe with minimal side-effects [16]. Moreover, phytochemicals derived drugs also possess wide therapeutic window than the conventional inorganic chemicals based drugs. This fact indicates that these drugs exhibit more therapeutic response upon administration than the possible adverse effects.

Therefore, in accordance to this scenario, cinnamon extracts were taken into consideration for this study, since, cinnamon has also been used for centuries as herbal medicine against various infections. It possesses different compounds which potentially exhibit antibacterial characteristics. These compounds include *cinnamyl acetate*, *benzyl benzoate*, *cinnamaldehyde* and *eugenyl acetate*, among others [17]. Many experimentations have been performed in different scenarios to elucidate their effectiveness against certain diseases and infections [18, 19]. These phytochemicals have been shown to inhibit bacteria by inducing the cell membrane degeneration, modifying the lipid profile, blocking ATPases, and inhibiting the cell division [20–24]. Due to such significant anti-bacterial features of these compounds, the major cinnamon compounds were selected to explore their efficacy against the targeted virulent proteins of *H*. *pylori*.

Hence, the structural modelling of these proteins was initially performed to predict the three dimensional (3D) structures of the virulent proteins. For the quality assessment of the predicted protein models, the Ramachandran plots were generated which affirmed their good quality. Afterwards, these proteins were subjected for docking analysis. In this regard, seven different cinnamon compounds were selected including cinnamyl acetate, benzyl benzoate, cinnamaldehyde, eugenol, etc. While, for docking purpose, the structure data format (.sdf) files of these compounds were retrieved from ChEBI database (https://www.ebi.ac.uk/chebi/). Subsequently, the docking analysis was performed in molecular operating environment (MOE) by employing the induce-fit or flexible docking model. Resultantly, two compounds i.e., *cinnamyl acetate* and *benzyl benzoate* were screened out to be the potential drugs. For more insights of these structural interactions, molecular dynamics (MD) simulations were performed in order to assess the structural stability and intramolecular motions of the amino acid residues of respective virulent proteins in the protein-ligand complexes. The findings suggested that all of the docked complexes possessed significant structural stability. Moreover, the pharmacokinetic perspectives of *cinnamyl acetate* and *benzyl benzoate* were also explored to comprehend their medicinal characteristics. This analysis was performed on two different online servers that are SwissADME (www.swissadme.ch/) and ADMETLab 2.0 (https://admetmesh.scbdd.com/). The collective results suggested that *cinnamyl acetate* and *benzyl benzoate* could be the potential drugs/inhibitors against the *virB4*, *virB8* and *virB9* proteins of *H*. *pylori* because they were found to possess significant pharmacokinetic properties.

## Materials and methods

### Amino acid sequence retrieval

In order to perform the structural modelling of *virB4 (accession no*.: *AIA98891*.*1)*, *virB8 (accession no*.: *ACJ07620*.*1)* and *virB9 (accession no*.: *ACF17785*.*1)* proteins of *H*. *pylori*, their amino acid sequences were required. These amino acid sequence were retrieved from National Centre for Biotechnology Information (NCBI) (https://www.ncbi.nlm.nih.gov/). These sequences were retrieved in.*fasta* format, since the subsequent tools to be employed for structure prediction supports this particular format of the input sequence.

### Structure prediction

The structural modelling was performed by SWISS-MODEL server (https://swissmodel.expasy.org/) [25]. The retrieved amino acid sequences were subjected for models prediction. This server follows the comparative modelling approach for structure prediction. The given query sequence is aligned with the template sequence of the experimentally predicted structures, stored in the database. On the basis of the sequence identity and alignment scores of target and template, various models are predicted. For the selection of the most appropriate predicted protein structure, various parameters were considered, e.g., sequence identity percentages, coverage and GMQE score. These parameters are quite crucial while selecting the best predicted model. The sequence identity percentage indicates the score of target-template sequence alignment and is the basis of comparative modelling. Therefore, the models with highest sequence identities and coverage were opted. This approach of model selection was followed in order to ensure the precise structural conformations of the predictive models. Moreover, the GMQE is the score of global model evaluation in which the score range of 0 to 1 is considered, with the higher number indicate good expected quality. While, 0.5 GMQE score indicates that at least 50% of the target sequence exhibited the coverage while alignment with the template sequence. The predicted structures were then downloaded in protein data bank (.*pdb*) format to proceed them for models evaluation and docking analysis.

### Quality validation of predicted structures

PROECHECK, which is available on UCLA-DOE LAB integrated server (https://saves.mbi.ucla.edu/) was employed to evaluate the quality of the predicted protein structures. This server generates the Ramachandran plot which is comprised of four different quadrants. Each one of them represents the most favorable region, generously allowed and disallowed regions, denoted by red, yellow and white colors, respectively. The allocation of amino acid residues of the given protein in any of these regions, is based upon the evaluation of their stereo-chemical properties and torsional angles. Moreover, the steric hindrances of the given protein models are also considered in this aspect. Based upon the overall calculations of these parameters, the residues are allocated in any of the regions. Therefore, the.*pdb* files of the proteins under study, were uploaded one after the other for this particular estimation. Ligands library prediction.

### Ligands library preparation

The major cinnamon compounds which have been reported to show therapeutic activity were selected after the literature review [7] for this research work. Resultantly, seven different compounds were enlisted. The molecular structures of these compounds were retreieved in structure-data format (.*sdf*) files from the ChEBI database (https://www.ebi.ac.uk/chebi/) [26]. Later, the library of these ligands were constructed for subsequent docking analysis. Molecular docking analysis.

### Molecular docking analysis

Molecular docking is the analysis of how two or multiple molecular structures interact with each other in different conformations. Moreover, their extent of interaction is also evaluated in this particular process so that insights regarding their binding affinities for each other, could also be elucidated. Molecular operating environment (MOE *version 2015*.*10*) was incorporated to perform the docking analysis in this study. This tool is already equipped with AMBER force field to perform various types of interactions of the given structures. The.*pdb* files of *virB4*, *virB8* and *virB9* proteins were subjected one-by-one in their respective docking performance. The ‘site finder’ function of MOE was employed in order to predict various active sites of proteins for docking. Before initiating the docking, it was highly crucial to opt the docking interaction model. For this purpose, the induce-fit refinement model was selected. Since, induce-fit approach allows a flexible conformation for the interacting structures, therefore, this method was selected so that the most appropriate binding modes could be produced. The London dG scoring method was chosen for this particular analysis. The functional form of London dG scoring method is shown below in the form of an equation:

$$\Delta G=c+E_{flex}+\sum_{h-bonds}c_{HB}f_{hb}+\sum_{m-lig}c_{M}f_{M}+\sum_{atoms\;i}\Delta Di$$

The number of different poses of each of the incorporated compound was set to more than one, so that different docking conformations could be attained to ensure the best docking orientation. The energy scores (S) and root mean square deviation (rmsd) values were targeted to evaluate the docking results. While, the docking outputs were kept for further investigations.

### Visualization and post-processing of docked complexes

The docked complexes were saved in protein data bank (.pdb) format for further processing. To have clear insights regarding the interacting residues of the proteins with the respective ligands, BIOVIA Discovery Studio Visualizer *ver*. 2021 was employed. This tool was specifically utilized to clearly infer the two-dimensional and three dimensional representations of the interacting residues in the respective docked complexes. Molecular dynamics (MD) simulations.

### Molecular dynamics (MD) simulations

The molecular dynamics (MD) simulations provides insights regarding the physical movement of the atoms or residues in a protein or any other molecule over time [27]. It is a method in which the physical conditions are applied to understand the ligand-protein interaction, the structural stability of their bound complex and the effect of various physical conditions on these molecules in a computational environment. This method also enables to analyze the binding conformations of two different structures in a more accurate form. In the current scenario, the respective docked complexes of virB4, virB8 and virB9 proteins were subjected to molecular dynamics simulations, to better reveal the binding interactions between the respective ligands and proteins under study. For this purpose, iMODS server (https://imods.iqfr.csic.es/) was employed [28]. This specific server provides multi-scale simulations of the given docked complexes. The results generate six different maps which enable to comprehend the interactions of protein and ligand from different perspectives. These maps include deformability, normal mode analysis (NMA), eigenvalue, variance, co-variance and elastic network model matrices. The deformability map computes the atomic displacements gradient which is obtained from different modes at various atomic indices. The high peaks represent the flexibility regions within the interacted residues. The NMA or Bfactor graph can also be correlated with this map for inferring the flexible states of the amino acid residues in accordance to their equilibrium position. Eigenvalues and variance maps give illustrations regarding the structural stability of the given complexes. The higher eigenvalues indicate that more energy will be required to deform the docked complex which signifies its higher structural stability. The co-variance matrix provides details about the correlated, uncorrelated and anti-related motions of the amino acid residues. Whereas, the elastic network model map describes the motion stiffness of the interacting residues based upon the intensity of the gray color in the map. In our study, this MD simulations method provided the significant outcomes regarding the *virB4-cinnamyl acetate*, *virB8-benzyl benzoate* and *virB9-cinnamyl acetate* complexes. In this context, the default parameters were followed to compute the required simulations.

### Pharmacokinetics analysis of cinnamon compounds

Pharmacokinetics is actually analyzing the body’s response to the drugs and the way how the body affects the drugs beings administered. This analysis is based on four different factors which include absorption, distribution, metabolism and excretion of the drugs [29]. This work also aimed to explore all such properties of the screened out ligands. For this purpose, water solubility, gastrointestinal (GI) absorption, skin permeability among other absorption properties were analyzed. Similarly, in context of distribution, plasma protein binding (PPB) and volume of distribution (VD) of the targeted compounds were taken into consideration. While, inhibition against certain crucial cytochrome enzymes including CYP1A2, CYP2C9 and CYP2D6 among others, in scenario of metabolism was analyzed. Whereas, total clearance and half-life of the drugs were explored to understand the excretion of the selected compounds. The toxicity analysis was also crucial in this aspect so that any possible toxic features of the ligands under study could also be estimated. Therefore, skin sensitization, eye corrosion, respiratory toxicity and acute toxicity rule were computed in this regard. All these properties of the docked cinnamon compounds were analyzed by incorporating the SwissADME (http://www.swissadme.ch/) and ADMETlab *version 2*.*0* (https://admetmesh.scbdd.com/) servers [30, 31]. The structure data format (.*sdf*) files of the targeted ligands were given as input in SwissADME server while, the SMILES format of these ligands were incorporated in the ADMETlab server 2.0 for the required analysis. The SMILES is actually the editable chemical formula in text form which is usually given as input data for such analyses. This overall analysis was quite crucial in terms of exploring the medicinal and drug-based characteristics of cinnamon extracts. On the basis of the results produced, it was inferred whether the targeted ligands/compounds would possess the affinity for real-time pharmacologically formulation or not.

## Results

### Structural modelling of virB4, virB8 & virB9 proteins

**Fig 1:**
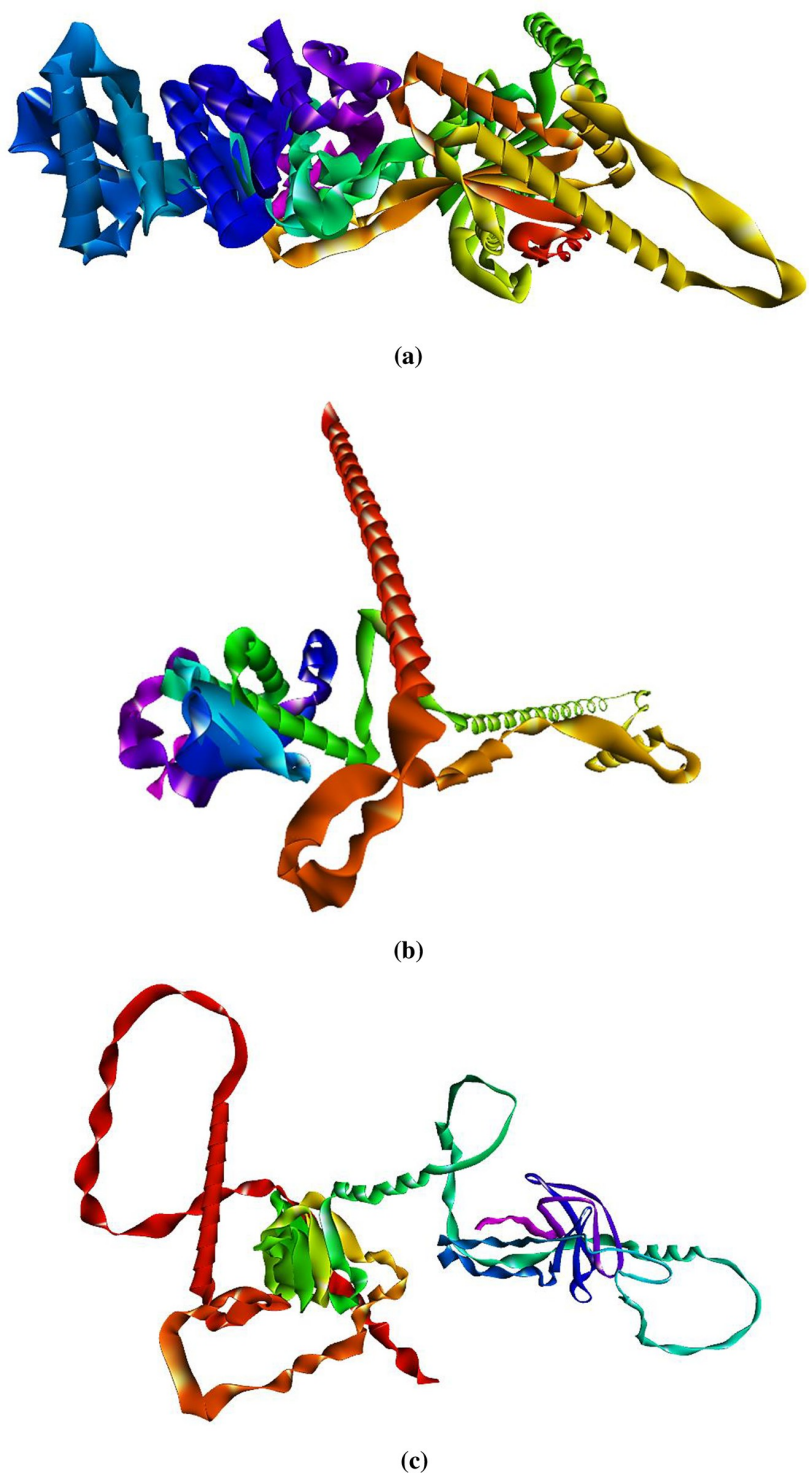
Predicted three dimensional models of virulent proteins of *H*.*pylori*. **(a)** virB4 protein, **(b)** virB8 protein, **(c)** virB9 protein.

The three dimensional structure of *virB4*, *virB8* & *virB9* proteins of *H*. *pylori* were predicted through comparative modelling approach. SWISS-MODEL was employed in this context, as discussed above. The sequence identity, coverage and GMQE scores were mainly considered in order to select the predicted models. The sequence identity and GMQE scores of *virB4* protein model were calculated as 96.48% and 0.87, respectively. While, that of *virB8* protein model 97.43% of the sequence identity and 0.63 GMQE were the resultant scores. The scores of these parameters were considerably appreciable to ensure the good quality of *virB9* protein as well, i.e., 89.71% sequence identity and 0.56 GMQE score. Whereas, the coverage in the results of all of the predicted models was maximum i.e., 100%. All of the resultant parameters explained previously along their scores, have also been included in the Fig 1 in S1 File. The structure of *virB4* protein seemed to be quite complex in terms of compact conformation of the protein chains than *virB8* and *virB9*. The quality of these proteins were further estimated by the subsequent Ramachandran plot. The three dimensional (3D) models of all the proteins under study are represented in the Fig 1.

### Quality evaluation of the predicted models

**Fig 2:**
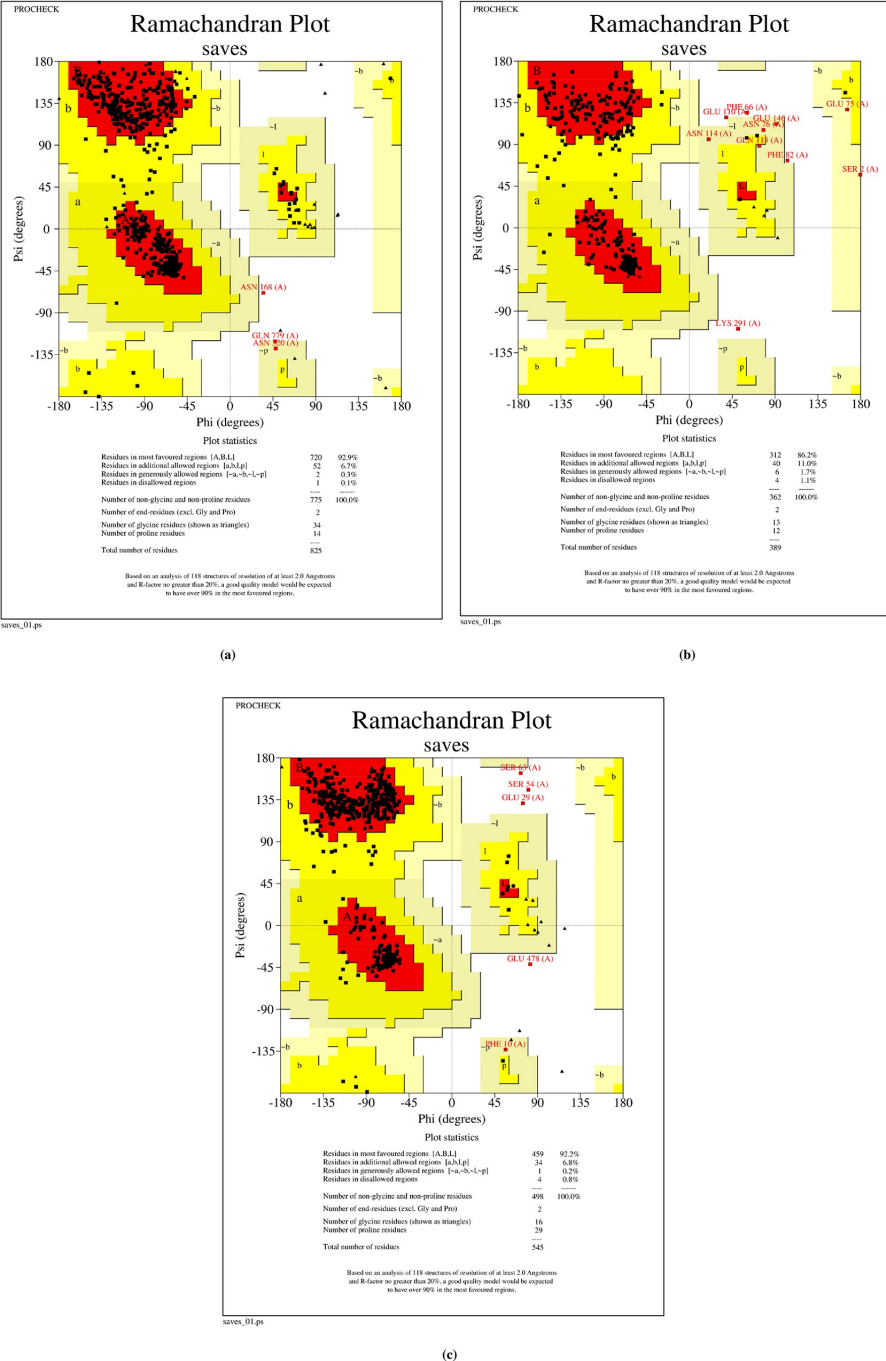
Ramachandran plots of predicted protein models. **(a)** *virB4* protein, **(b)** *virB8* protein, **(c)** *virB9* protein. All of the plots represented that the majority of the amino acid residues of these three proteins lied in the favorable region depicting the good quality of the predicted models.

The quality of predicted 3D models of *virB4*, *virB8* and *virB9* proteins were evaluated by Ramachandran plot, available on PROCHECK server. The Ramachandran plot of *virB4* protein suggested that 92.9% of the amino acid residues lied in the favorable region while the rest of the residues were allocated in additionally allowed and generously allowed regions. Only 0.1% of the residues were allotted in disallowed portion of the plot. Whereas, the required results of *virB8* proteins presented that 86.2% and 11% of the residues were found in favorable and additionally allowed regions, respectively. Only 1.7% and 1.1% of the residues of this proteins were respectively found in generously allowed and disallowed regions of this plot. Lastly, the third protein under study i.e., *virB9* showed a bit similar Ramachandran favored region score to that of *virB4* protein. Its 92.2% of the residues were allocated in the favorable region. While, 6.8%, 0.2% and 0.8% of the amino acid residues lied in additionally allowed, generously allowed and disallowed regions of the Ramachandran plot, respectively. Upon conclusive analysis of the results, it was inferred that the predicted protein models were of good quality. The results of Ramachandran plot of these three proteins are demonstrated below in Fig 2.

### Molecular docking and virtual screening

The predicted 3D structures were then incorporated for molecular docking analysis to interact with the cinnamon compounds. The objective of this interaction was to screen out the potential compounds which would show good binding affinities with *virB4*, *virB8* and *virB9* proteins. The structure data format (.sdf) files of all the cinnamon compounds were retrieved from ChEBI database. The following Table 1 comprises of required details of all these extracts including their names, ChEBI i.ds and structures.

**Table 1:** Details of Cinnamon extracts along their structures.

CHEBI ID	Cinnamon Extracts
**CHEBI: 16731**	Cinnamaldehyde
**CHEBI: 4917**	Eugenol
**CHEBI: 27386**	Cinnamic acid
**CHEBI: 10357**	Caryophyllene
**CHEBI: 41237**	Benzyl benzoate
**CHEBI: 17580**	Linalool
**CHEBI: 31402**	Cinnamyl acetate

**Fig 3:**
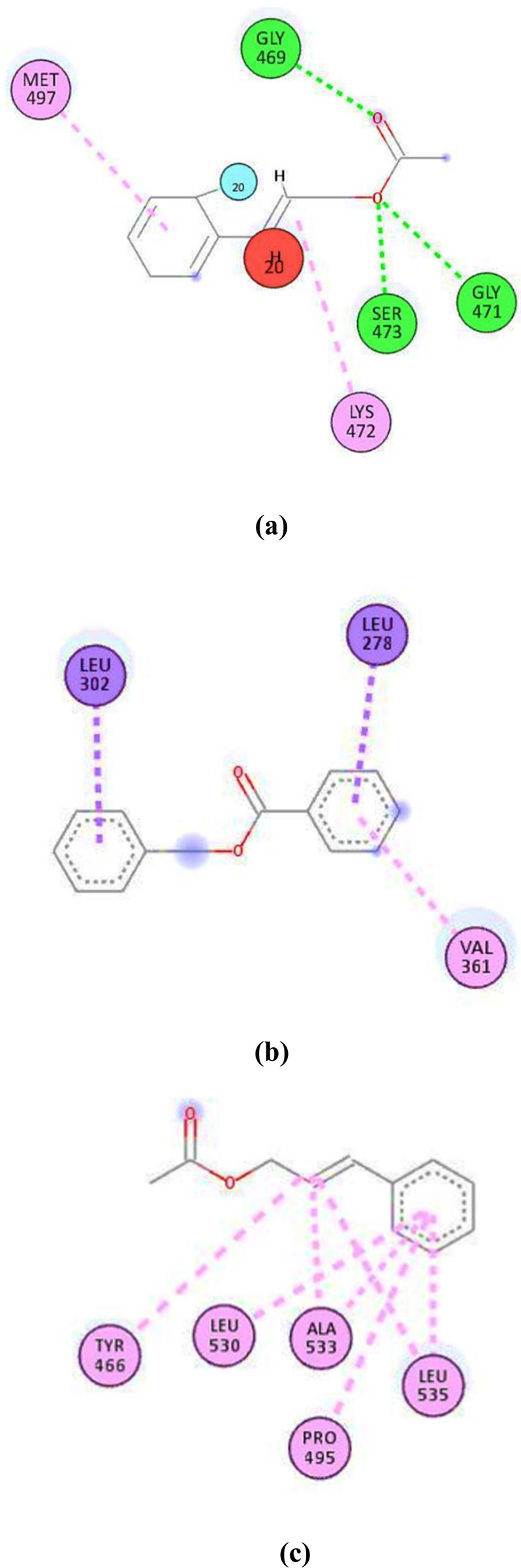
Two dimensional representations of the docked complexes. **(a)** *virB4-cinnamyl acetate* complex, (**b)** *virB8-benzyl benzoate* complex, **(c)** *virB9-cinnamyl acetate* complex.

**Fig 4:**
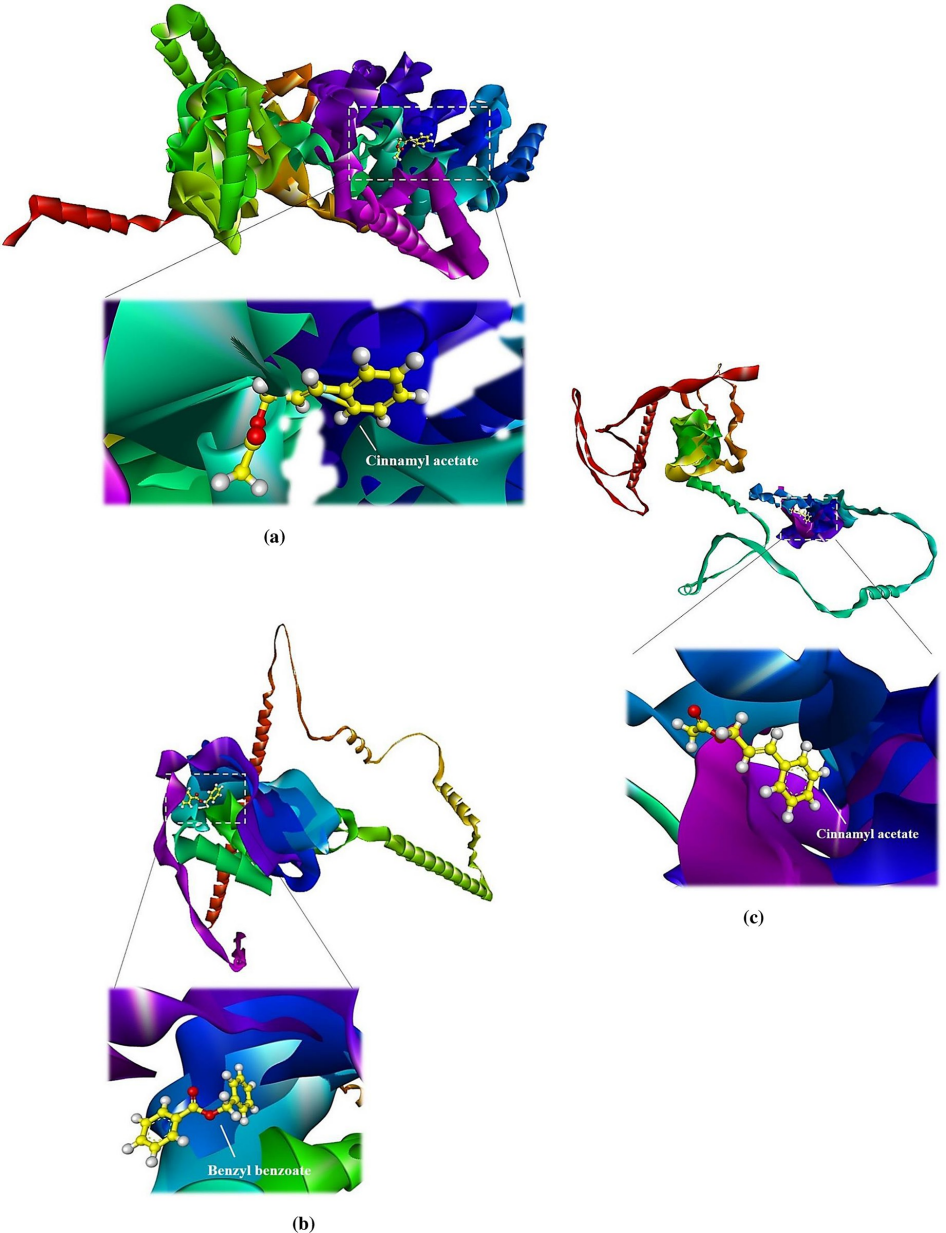
Three dimensional (3D) representations of the docked complexes. **(a)** *virB4*-*cinnamyl acetate* docked complex, **(b)** *virB8*-*benzyl benzoate* complex, **(c)** *virB9*-*cinnamyl acetate*.

The induce-fit model was opted to perform the docking analysis. This type of docking is also known as flexible docking [32] because it enables a degree of flexibility in protein structure. This structural flexibility enables the given protein to accommodate and interact with the binding ligand to its maximum extent which ultimately leads to the formation of a stable docked complex. Initially, the *virB4* protein were docked against all the above mentioned compounds. In this analysis, *cinnamyl acetate* was found to show the best docking results among other ligands. The binding energy (S) and root mean square deviation (rmsd) scores were calculated as-4.8161 kcal/mol and 1.87, respectively. This S-score was the best among all of the other docked complexes including their different docked poses. Furthermore, it was observed that *cinnmamyl acetate* was quite occupied inside the interacting binding site of the *virB4* protein. The interacting residues of this region were specifically the serine, methionine, lysine and glycine. Whereas, the *virB8* protein exhibited bit different docking results. This protein particularly interacted with *benzyl benzoate*, showing -4.7916 kcal/mol and 1.65 energy and rmsd scores, respectively. However, the *virB9* protein displayed the similar docking results to *virB4* protein in terms of showing the good binding affinity with the *cinnamyl acetate*. But, the binding energy and rmsd scores were -5.5914 kcal/mol and 1.17, respectively, which were different from that of *virB4*-*cinnamyl acetate* docked complex. The Fig 3 illustrates the two dimensional orientations of *virB4*-*cinnamyl acetate*, virB8-benzyl benzoate and virB9-cinnamly acetate docked complexes. While, the Fig 4 comprises of 3D visualization layouts of all the docked complexes.

**Fig 5:**
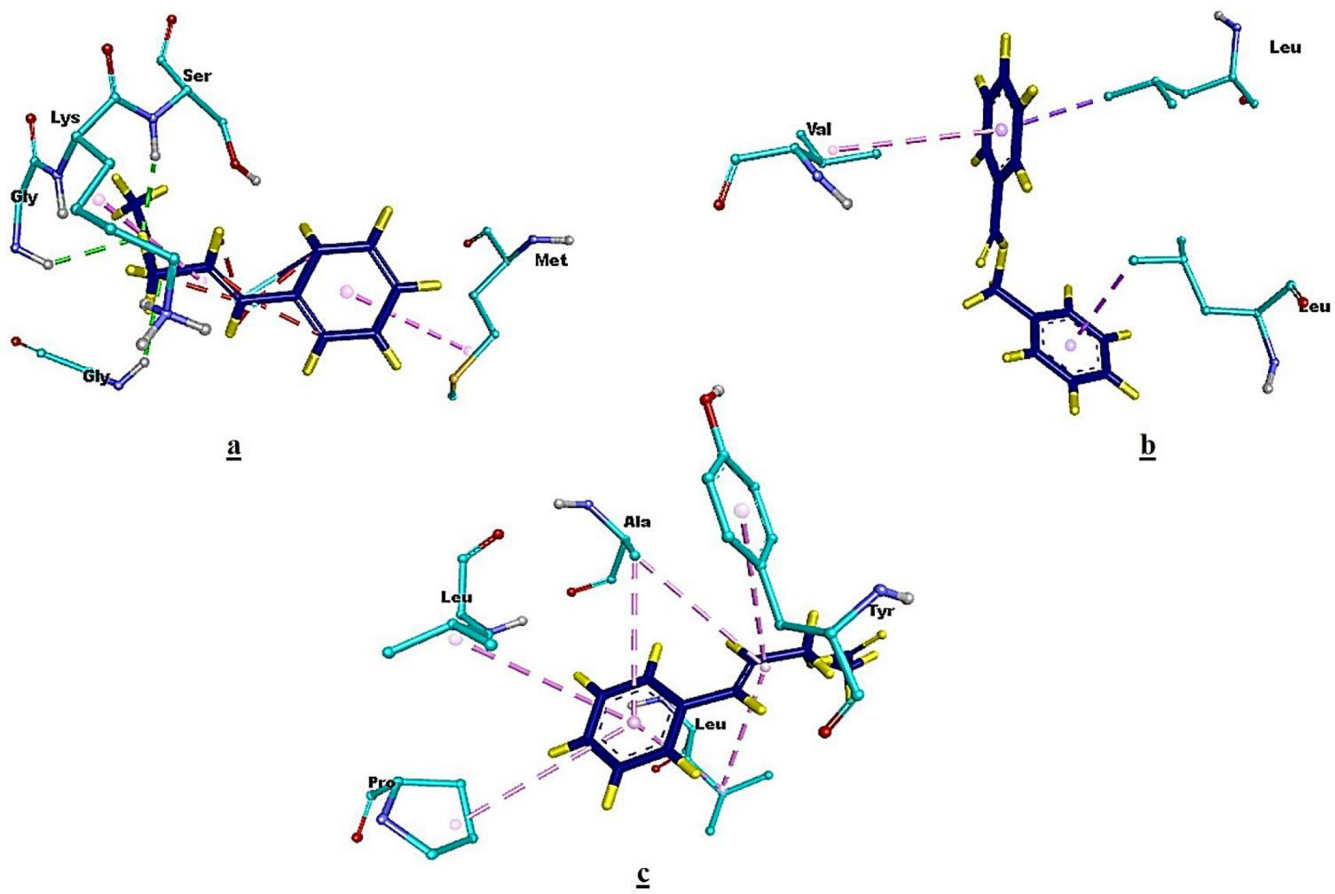
3D Illustration of respective interacting atoms between the docked proteins and their respective compound. **(a)** interacting residues of *virB4*-*cinnamyl acetate* docked complex, **(b)** *virB8*-*benzyl benzoate* interacting residues, **(c)** interacting residues in *virB9*-*cinnamyl acetate* docked complex.

Furthermore, to get more insights regarding the docked complexes, the 3D interaction results were also produced which clearly displayed the interaction of the docking residues with their respective ligands. In *virB4-cinnamyl acetate* docked complex, the MET497, GLY469, SER473, GLY471 and LYS472 were the interacting protein residues. Whereas, in the docked complex of *virB8-benzyl benzoate*, the interacting residues included, LEU302, LEU278 and VAL361. However, the interacting amino acid residues in the *virB9-cinnamyl acetate* complex included, TYR466, LEU530, ALA533, PRO495 and LEU535. The three dimensional representations of all the docked complex to illustrate such interactions are represented in the following Fig 5.

### Molecular dynamics (MD) simulations of docked complexes

**Fig 6:**
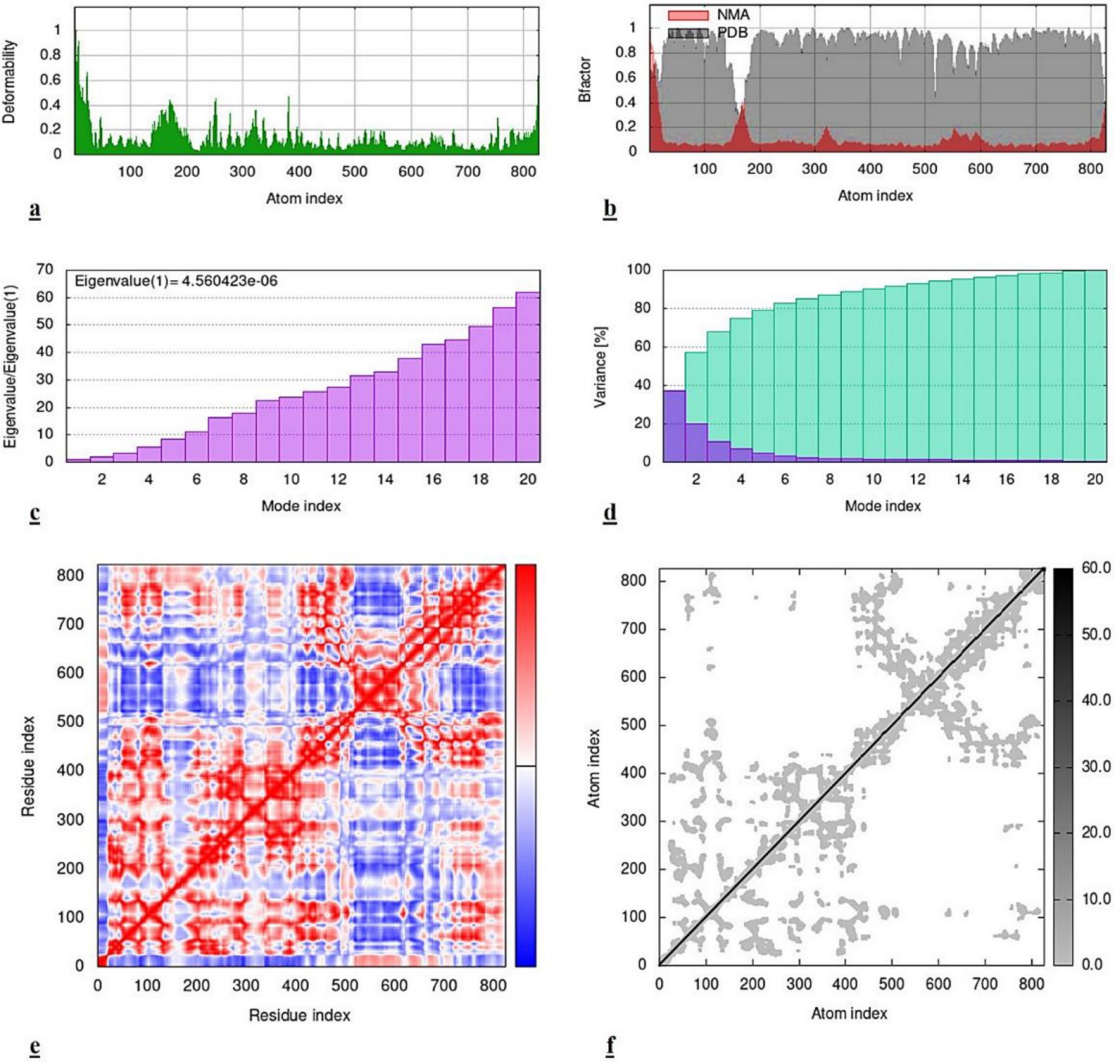
MD simulation results of *virB4-cinnamyl acetate* complex. **(a)** Deformability map, **(b)** Bfactor, **(c)** Eigenvalue, **(d)** Variance, **(e)** Co-variance, **(f)** Elastic network model.

The molecular dynamics and simulations provided better insights of the docking analysis. It enabled the actual discernment regarding the internal coordinates of two docked entities. Various parameters were considered in this regard in order to interpret the results. In the Fig 6, the first map depicts various peaks to indicate the degree of deformability in the docked structure. Variable levels of deformability in the *virB4* protein were observed at various residual sites. But, its higher degrees were witnessed at the initial atom indices. This fact was further validated by the B-factor graph which also exhibited similar fluctuations. This particular graph focuses on the normal mode analysis of the docked complex. In our study, significant levels of difference were observed at different atomic intervals in *virB4*-*cinnamyl acetate* complex. Furthermore, the map presenting the eigenvalues indicates the energy required to deform a molecular structure. This fact reflects that if there would be the lower values then the deformation in the docked complex would be easier. In the current scenario, the eigenvalue was 4.56e-06 which indicated the appreciable structural stability of protein-ligand complex. Contrariwise, the variance is the inverse phenomena of eigenvalues. This graph explicitly showed the cumulative and individual variance values of the amino acid residues. While, the co-variance graph directed towards three major factors of the *virB4* protein and *cinnamyl acetate* interaction including, the correlated (red color), uncorrelated (blue color) and anti-correlated (white color) motions of the interacting residues. In the following figure, it can be witnessed that the region of the co-variance map between 0 to 500 residue indices along both X and Y-axis, displayed more correlated motions of the docked *virB4-cinnamyl acetate* complex. Though, some of the uncorrelated and anti-correlated regions of interaction were also detected at various sites. The last map in the figure below, shows the elastic network model which focuses on the extent of flexibility in the target structure. This structural flexibility can be assessed on the basis of the intensity of gray color. In the current scenario of *virB4-cinnamyl acetate* complex, the results proposed that the correlated residues which we also discussed previously, possessed significant flexibility in its structure. This ultimately indicated that *virB4 protein* showed good interactions with *cinnamyl acetate* and also, their docked complex was structurally stable.

**Fig 7:**
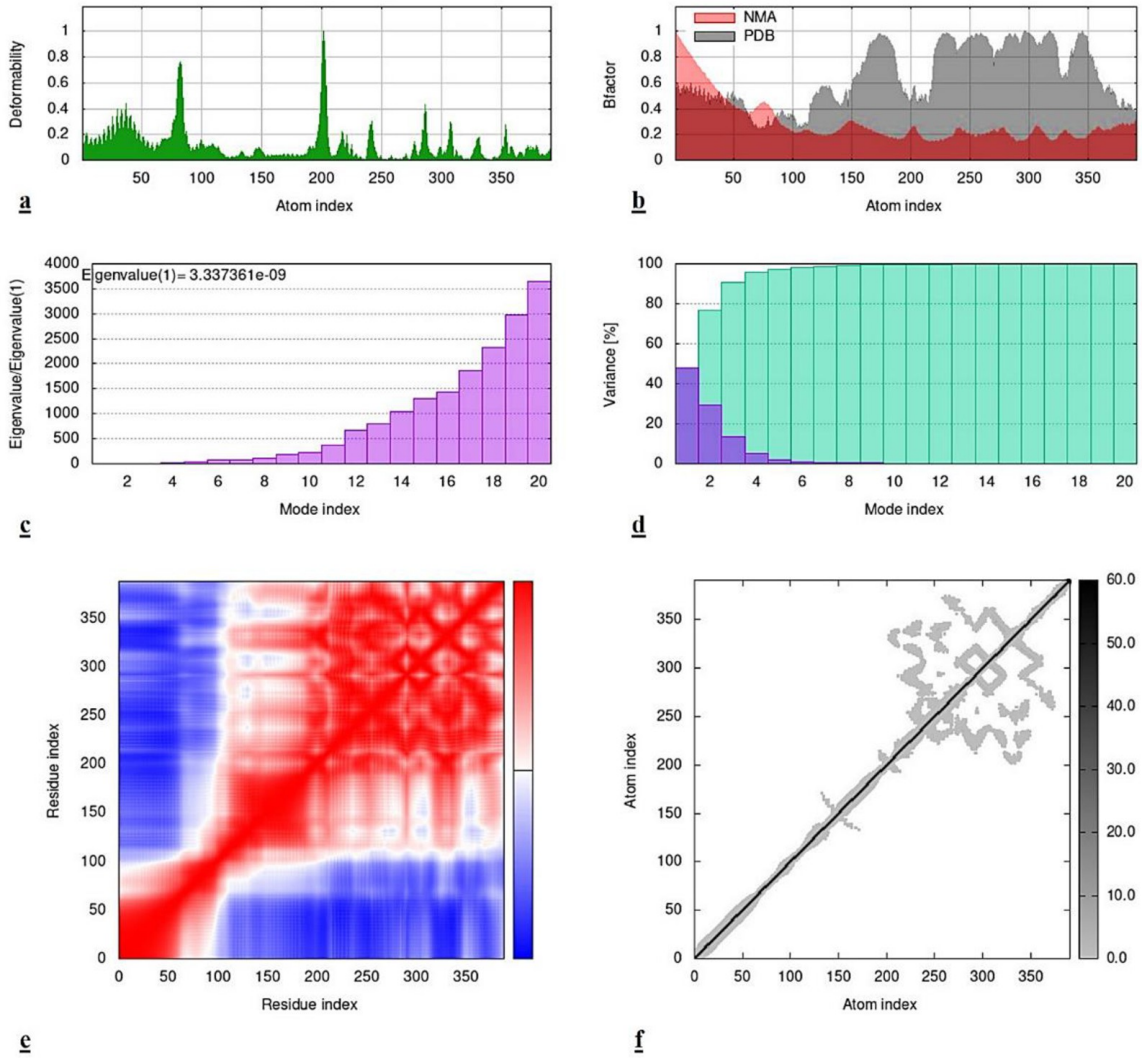
Results of MD simulations of vir*B8-benzyl benzoate* complex. **(a)** Deformability map, **(b)** Bfactor, **(c)** Eigenvalue, **(d)** Variance, **(e)** Co-variance, **(f)** Elastic network model.

Comparatively, the MD simulations of *virB8-benzyl benzoate* complex displayed a bit different set of results. The higher deformability hinges in *virB8 protein* were noticed at the integral residual sites than at the initial or terminal regions. While, significant levels of difference between the peaks of NMA and PDB were observed in Bfactor calculations. The obvious evaluation of interaction quality was analyzed by the eigenvalue and variance graphs. Both of these parameters suggested that the interaction was a bit deficient in quality because the calculated eigenvalue i.e., 3.33e-09 and variance, both were not that significantly good as those observed in *virB4-cinnamyl acetate* results. The results of co-variance were also different in this context. More correlated motions were observed at 100–350 residual indices than at the initial ones. However, the elastic network model directed that there were higher levels of flexibility in the interacting residues of the target protein i.e., *virB8*. All of these results can be visualized in the following Fig 7 in which each graph is representing the respective phenomena.

**Fig 8:**
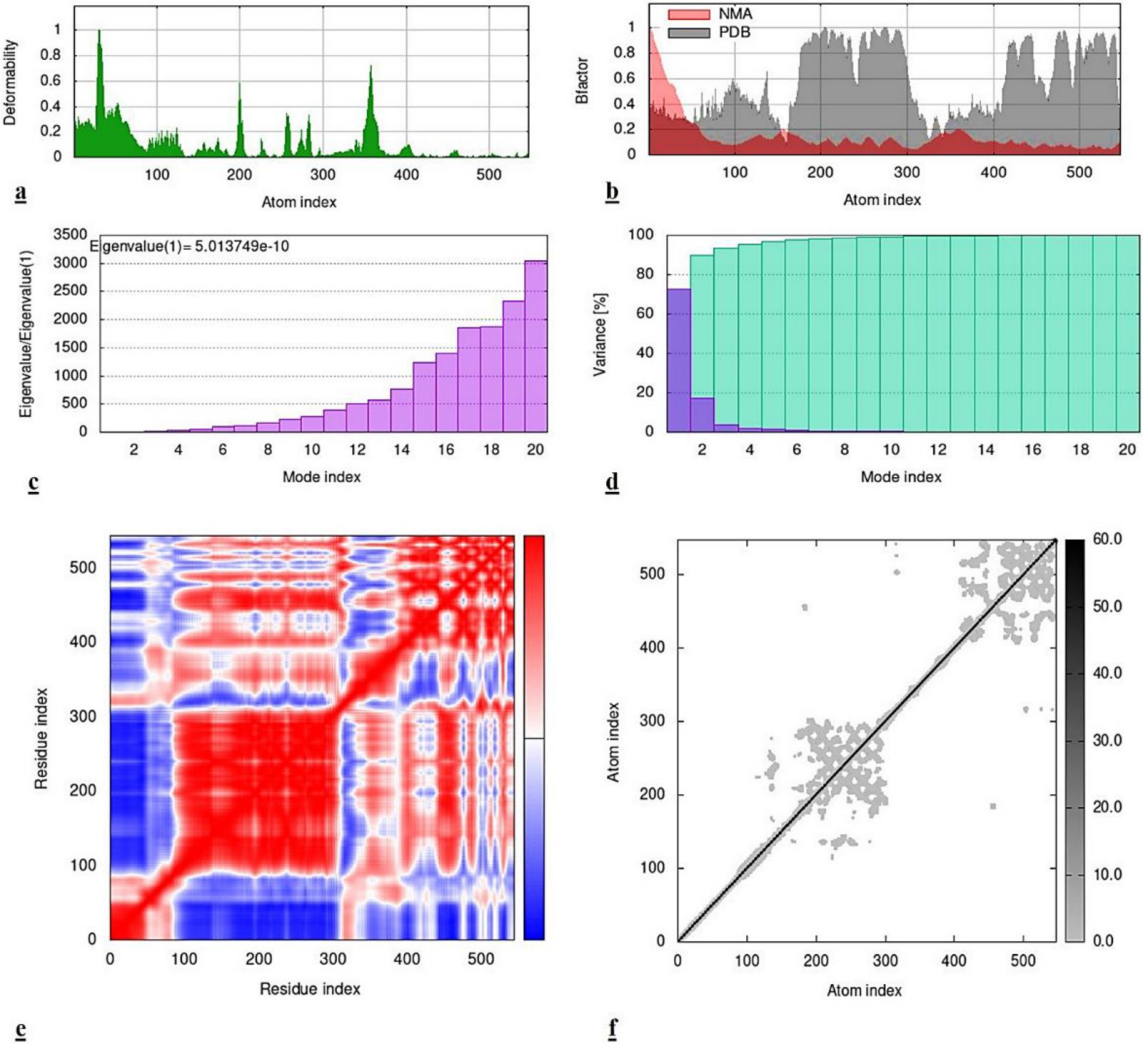
MD simulation results of *B9-cinnamyl acetate* complex. **(a)** Deformability map, **(b)** Bfactor, **(c)** Eigenvalue, **(d)** Variance, **(e)** Co-variance, **(f)** Elastic network model.

Furthermore, this particular analysis of virB9-cinnamyl acetate interestingly suggested somehow similar results to that of *virB4-cinnamyl acetate* complex. The higher degree of deformability was observed at the initial residual sites than the rest ones. The Bfactor map represented more difference between NMA and PDB at interior and terminal sites. Moreover, the calculated eigenvalue was 5.01e-10 which proposed that the deformability in this docked complex would not be easy and more energy would be required to do so, which eventually affirmed the significant structural stability of *virB9*-*cinnamyl acetate* complex. The variance graph also conversely complied with the results of eigenvalues, affirming again that the interaction results were significantly good. While, the correlated motions were abundantly observed at initial 300 residual indices. Although, the uncorrelated and anti-correlated were also witnessed in the subsequent regions. Lastly, the elastic network model revealed that all of the interacting residues of the *virB9 protein* possessed noticeable flexibility. All of these results are presented below in the Fig 8. However, the inclusive results of MD simulations of all the three docked complexes suggested a conclusion that *cinamyl acetate* presented better interaction than the *benzyl benzoate*.

### ADMET analysis of *cinnamyl acetate* and *benzyl benzoate*

**Fig 9:**
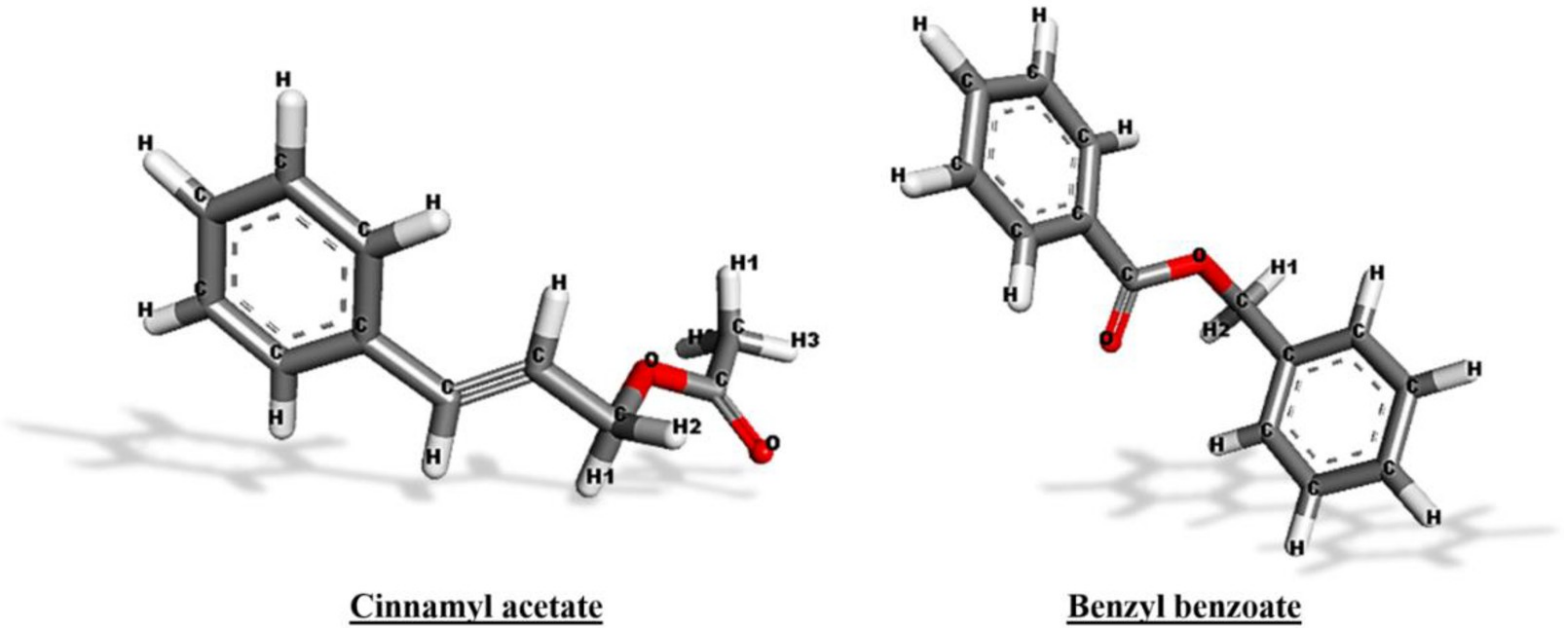
Molecular structures of *cinnamyl acetate* and *benzyl benzoate*.

The results of molecular docking and MD simulations screened out two compounds i.e., *cinnamyl acetate* and *benzyl benzoate*. Both of these ligands demonstrated comparatively better interactions than the rest of the compounds. Hence, it was crucial to explore their ADMET properties to unveil their medicinal or drug-likeness perspectives. The molecular structures of both of these compounds are represented below in Fig 9.

**Fig 10:**
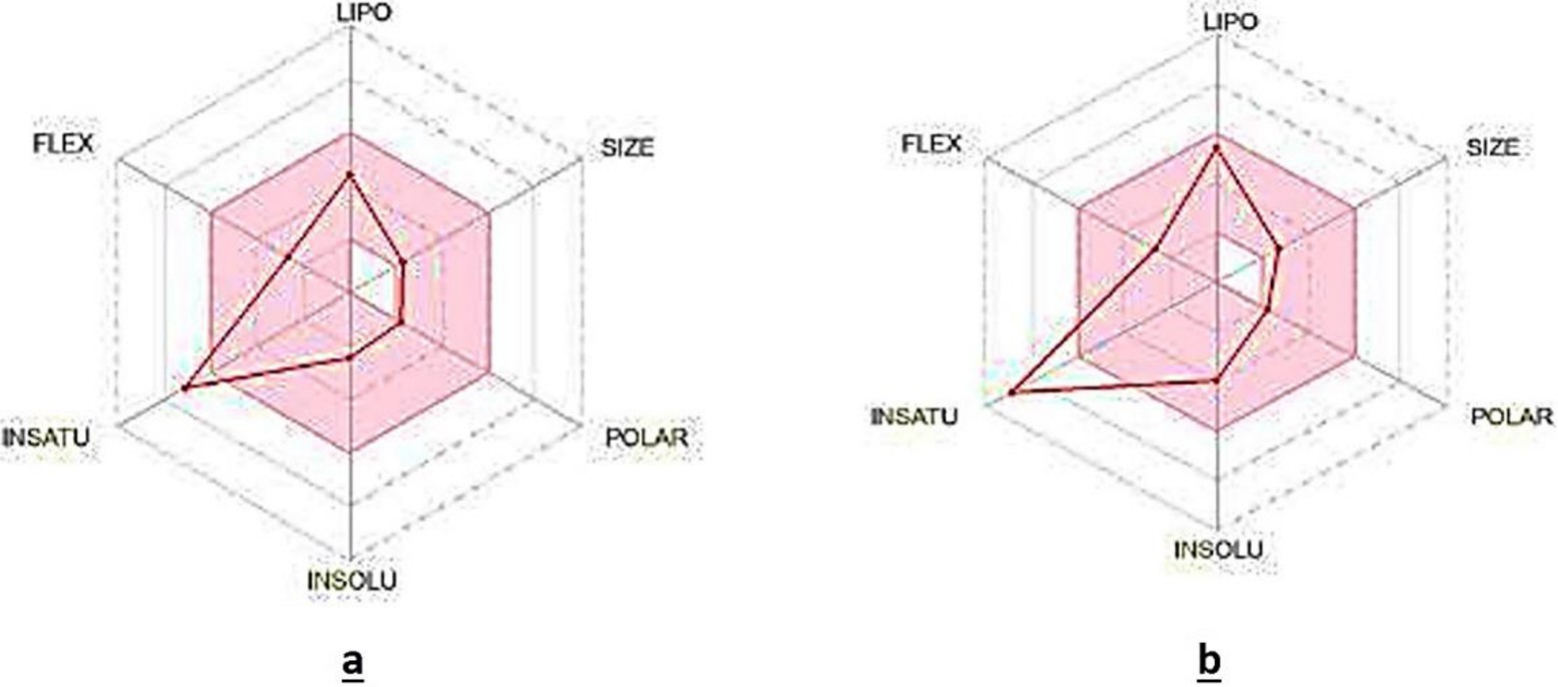
Bioavailability radars. **(a)** *Cinnamyl acetate*, **(b)** *Benzyl benzoate*. Both of these radars represented various characteristics of the drugs under study which include, FLEX: Flexibility, LIPO: Lipophilicity, INSATU: Insaturation and INSOLU: Insolubility.

Both of these drugs were subjected initially to SwissADME to infer fundamental drugs properties. The following Fig 10 highlights some of the crucial features of *cinnamyl acetate* and *benzyl benzoate*. According to the bioavailability radars in this figure, both of these compounds were showing a degree of *insaturation*, which suggested that they would be more reactive.

The collective results of SwissADME and ADMETlab 2.0 highlighted multiple pharmacokinetic aspects. Initially, the physiochemical properties were evaluated which focused on important parameters including the molecular weight, number of heavy atoms, fraction Csp3, number of acceptor and donor hydrogen bonds and topological polar surface area (TPSA). SwissADME indicated that both of the compounds completely complied with the required drug likeness parameters except the molecular weight. It suggested that the computed molecular weight values were slightly higher. While, the rest of the computed parameters showed valuable results including the molar refractivity and TPSA scores. These results are summarized in the following Table 2.

**Table 2:** Physiochemical properties of *Cinnamyl acetate* and *benzyl benzoate* predicted by SwissADME and ADMETlab.

PARAMETERS	CINNAMYL ACETATE	BENZYL BENZOATE
*Molecular weight*	176.21 g/mol.	212.24 g/mol.
*Number of heavy atoms*	13	16
*Number of aromatic heavy atoms*	6	12
*Fraction Csp3*	0.18	0.07
*Number of rotatable bonds*	4	4
*Number of Hydrogen bond acceptors*	2	2
*Number of Hydrogen bond donors*	0	0
*Molar refractivity*	52.24	62.21
*Topological polar surface area (TPSA)*	26.30 A^2^	26.30 A^2^

Since, pharmacokinetics is comprised of absorption, distribution, metabolism, excretion and toxicity analysis of drugs, therefore, their results are separately summarized within their respective sub-sections in Table 3. The absorption was estimated on the basis of multiple features predominantly, the water solubility and gastrointestinal (GI) absorption. Both of the compounds presented good results in these aspects. The skin permeability score was too low, proposing that *cinnamyl acetate* and *benzyl benzoate* would be almost impermeable to the skin. Other parameters are also given along their respective values in the following table. As far as, the distribution perspectives of these compounds were concerned, both of the compounds possessed high percentages of plasma protein binding (PPB). The calculated volumes of distribution (VD) of these compounds were 1.739 and 0.931, respectively. In context of metabolism, *cinnamyl acetate* did not show any inhibitory interaction with any of the isoforms of cytochrome P450 (CYP). However, *benzyl benzoate* represented inhibition for CYP1A2 and CYP2C19 indicating the fact that this compound will have lower levels of metabolism and may also lead to some unwanted effects. Furthermore, the total clearance and half-life values of these ligands proposed that they possessed appropriate excretion properties. The respective values can be observed in Table 3 in the sub-section of distribution. The toxicity levels of both of the compounds were also computed. Four major factors were taken into consideration in this regard including, the skin sensitization, eye corrosion, respiratory toxicity and acute toxicity rule. Both these drugs revealed variable extents of toxicity from, moderate to toxic and corrosive levels. *Cinnamyl acetate* were found to be toxic for skin but presented moderate toxicity for optical and respiratory system. Whereas, *benzyl benzoate* was found to be corrosive for eyes, toxic for respiratory tract and moderately toxic for integumentary system. But, both of the drugs did not violated the acute toxicity rule. However, the actual toxicity of these compounds may be validated upon *in-vivo* experimentations. The accurate therapeutic index and therapeutic window of both of these compounds could also be calculated in this particular aspect. Based on the overall findings, it can be anticipated that the prolong exposure to these drugs may cause the toxicity up to their certain calculated levels.

**Table 3:** ADMET properties of *cinnamyl acetate* and *benzyl benzoate*.

***Absorption***		
**Properties**	**Cinnamyl acetate**	**Benzyl benzoate**
Water solubility	Soluble	Moderately soluble
Gastrointestinal (GI) absorption	High	High
Skin permeability	-5.78 cm/s	-4.78 cm/s
P-glycoprotein substrate	No	No
Lipophilicity (Log *P*_*o/w*_)	2.33	3.25
***Distribution***		
**Property**	**Cinnamyl acetate**	**Benzyl benzoate**
Plasma Protein Binding (PPB)	82.850%	97.919%
Volume distribution (VD)	1.739	0.931
***Metabolism***		
**Property**	**Cinnamyl acetate**	**Benzyl benzoate**
CYP1A2 inhibitor	No	Yes
CYP2C19 inhibitor	No	Yes
CYP2C9 inhibitor	No	No
CYP2D6 inhibitor	No	No
CYP3A4 inhibitor	No	No
***Excretion***		
**Property**	**Cinnamyl acetate**	**Benzyl benzoate**
Total clearance	6.376	12.285
Half-life (T_1/2_)	0.863	0.803
***Toxicity***		
Skin sensitization	Toxic	Moderate
Eye corrosion	Moderate	Corrosive
Respiratory toxicity	Moderate	Toxic
Acute toxicity rule	0 alert	0 alert

The evaluation of these drugs was also figured out on the basis of certain rules which would assist in the assessment, either both them could be formulated as drugs in real-time or not. Interestingly, these compounds did not violated the Lipinski rule. In addition, their bioavailability score was 0.55 indicating that being the drugs, their circulation in the body would be noticeably virtuous. Moreover, the synthetic accessibility scores i.e., 1.98 and 1.44 of *cinnamyl acetate* and *benzyl benzoate* suggested that these drugs would be easy to develop for actual use. However, no leadlikeness values were found in the respective contexts of these drugs, depicting that no lead compound would be found as a drug which could be employed as a reference. Hence, these compounds may be proceeded further and evaluated to get even better insights regarding their drug-likeness. All of such important parameters are comprehensively summarized in the following Table 4.

**Table 4:** Other important parameters.

PARAMETERS	CINNAMYL ACETATE	BENZYL BENZOATE
*Lipinski rule*	Yes	Yes
*Bioavailability score*	0.55	0.55
*Synthetic accessibility*	1.98	1.44
*Leadlikeness*	No	No

## Discussion

Plant extracts, also known as phytochemicals are bioactive compounds which are produced in plants for their protection against different pathogens including viruses, bacteria and fungi [33]. Different plants, fruits and vegetables produce numerous number of such compounds. These compounds possess the potential to degrade the crucial cellular activities of pathogens [34]. In this aspect, one of the investigations have proposed that *S*. *rosmarinifolia* possessed not only anti-microbial properties but, the anti-inflammatory characteristics as well [35]. Afterwards, a comprehensive review regarding the pharmacological efficacy of *Mangifera indica* also illustrated that its extracts in variable proportions, have the anti-microbial features [36]. *Cymbopogon* spp. are also one of those plants which exhibited the anti-microbial and anti-oxidant attributes upon different investigations. According to a study, *C*. *proximus* and *C*. *citratus* were pharmacologically evaluated and then it was proposed that both of the plants could be the excellent antimicrobial sources and may also inhibit the *Malassezia* based infections [37]. Such significant attributes of all such plants make them plausible alternates to synthetic antibiotics and their extracts, to be the appealing candidates as drugs.

Cinnamon is also one of such plant which has also been evaluated in similar scenarios [38–40]. Its extracts especially, *cinnamaldehyde* and *eugenol* have been incorporated in multiple experimentations which have affirmed their antimicrobial potential [41–43]. Moreover, cinnamon was also found to be applied as antimicrobial herbal medicine for centuries [44]. The bioactive compounds of cinnamon include, *cinnamyl acetate*, *cinnamaldehyde*, *and benzyl benzoate* among others. The disease in which cinnamon compounds have been employed as a drug include, gastrointestinal (GI) and various parasitic infections [45]. These diseases are caused by a wide range of pathogenic microbes including fungal and bacterial strains. *H*. *pylori* is also one of the well-known pathogen causing a number of serious infections in human beings. The pathogenicity of this bacteria is determined by some virulent factors or proteins [46]. The previously published studies have majorly focused on CagA and VacA, the two major virulent proteins of *H*. *pylori* [46, 47]. But, one of such research works, published by D. Nammi *et al*. has highlighted other virulent factors from various isolates of *H*. *pylori*. These proteins were primarily the secretion system proteins and referred as *virB4*, *virB8* and *virB9* proteins [7]. In this particular study, these virulent factors were mainly targeted to evaluate the pharmacological efficacy of cinnamon extracts.

To reveal the effectiveness of cinnamon compounds against the above mentioned proteins, molecular docking, MD simulations and ADMET analyses were subsequently performed. Before proceeding towards these major analyses, the 3D structures of all three proteins were predicted. The SWISS-MODEL tool was employed to perform this task. The respective reported research works by Havva Esra Tütüncü & Yusuf Sürmeli and afterwards, by A. Baseer *et al*. have also followed the similar approach to predict the 3D models of the given proteins [48, 49]. However, Kannan I. *et al*. employed Modeller while predicting the three dimensional structure of SCCA protein in their respective work [50]. In the current study, the predicted structures of *virB4*, *virB8* and *virB9* proteins were then subjected to molecular docking analysis after structure quality assessment by their respective Ramachandran plots. In a comparative analysis of structural and functional characterization of TANK-Binding Kinase 1-Binding Protein by Sawal *et al*., the same method was opted to assess the stereochemical quality of the predicted protein structures [51].

Further, the evaluated 3D models of *virB4*, *virB8* and *virB9* proteins were then subjected to molecular docking analysis. The molecular operating environment (MOE) was incorporated which has also been widely employed in already reported studies [52–54]. We particularly selected the induce-fit model to perform this docking analysis. The published studies by Janani Prabaharan *et al*. and A. Mili *et al*. also followed the induce-fit docking method to attain the best conformation of docked complexes [55, 56]. In our study, it was found that *cinnamyl acetate* and *benzyl benzoate* represented comparatively good docking scores in their respective molecular docking analyses. *Cinnamyl acetate* interacted well with *virB4* and *virB9* proteins. Whereas, *virB8* exhibited appreciable docking interaction with *benzyl benzoate*. Such virtual screening approach has also been followed in the previously reported studies by Mouhcine M. *et al*. and Ejaz SA *et al*. [57, 58].Moreover, an i*n-silico* experimentation regarding the virtual screening of inhibitors was performed in order to unveil the potential of *Eclipta alba* against the glycoprotein of Hepatitis C virus (HCV). It was found that *oleanolic acid* can be used as an inhibitor against the protein under study [59].

Furthermore, to reveal more of the interaction motions and internal coordinates of the docked complexes in our context, MD simulations were incorporated. This particular analysis is performed to evaluate the structural stability of the docked complexes [60]. In current study, iMODS server was incorporated to perform this analysis so that diverse perspectives could be attained from such a multi-scale simulation approach. This tool has been widely incorporated in different research works. For example, M. Moharana *et al*. employed this particular method to assess the structural stability of the docked complexes in their respective work [61]. While, many previous studies regarding the *in-silico* vaccine designing have also utilized this online server for normal mode analysis and multi-scale dynamics studies [62–64].

Whereas, in the current work, these simulation results revealed that *cinnamyl acetate* associated docked complexes displayed comparatively better interactions as compared to the *virB8*-*benzyl benzoate* complex. Various parameters of the computed results were taken into consideration to evaluate this fact. These factors included the deformability, normal mode analysis (NMA), eigenvalues and elastic network model among others. *Cinnamyl acetate* presented significantly virtuous extent of motions of interaction under these parameters especially, deformability, eigenvalues and elastic network model. While, the *benzyl benzoate*-*virB8* complex although resulted with an appreciable levels of deformability hinges but the results of eigenvalues and elastic network model were not that significant. Various studies have also incorporated similar approach to elucidate the binding motions through MD simulation [65]. According to a reported research on the potential of chromone‑embedded peptidomimetics as inhibitors of SARS-CoV-2, it was found that Ch-p7 had the best binding results. The MD simulations were performed further in the similar fashion, which confirmed that the docked complex was structurally stable [66].

Lastly, to evaluate the *cinnamyl acetate* and *benzyl benzoate* from the pharmacokinetic perspectives, ADMET analysis was performed. Initially, the physiochemical properties were analyzed which estimated the crucial features including molecular weight, number of acceptor and donor H-bonds, molar refractivity and TPSA of the ligands under study (*cinnamyl acetate* and *benzyl benzoate*). Kazi M. Rana *et al*. and Akshay R. Yadav *et al*. have also investigated the pharmacokinetics of *Malvastrum coromandelianum* and cytidine analogs, respectively [67, 68] but, the ADEMT analyses in their work was not that extensive as we have explored almost all of the crucial perspectives of *cinnamyl acetate* and *benzyl benzoate* in this study. However, the ADMET analysis presented by Qaddir *et al*. while investigating different phytochemicals as potential dengue virus inhibitors, were significantly appreciable [69]. R. Muthukumar *et al*. and Sajia Islam *et al*. have also explored the pharmacokinetic properties of the drugs under consideration by employing the similar approach [70, 71]. Afterwards, we also calculated five major factors regarding the pharmacokinetics of *cinnamyl acetate* and *benzyl benzoate*. These parameters included absorption, distribution, metabolism, excretion and toxicity. Both of the drugs underwent various evaluations in context of all these parameters so that their ADMET properties could be elucidated in a better way. The collective results suggested that both of these cinnamon compounds followed majority of the drug-likeness properties. This study would help to set down the foundations for in-vitro experimentations to analyze the efficacy of these compounds against *H*. *pylori* virulent proteins. Moreover, the specified analyses employed in this work could also provide directions for performing research work against other pathogens as well.

## Conclusion

This study primarily aimed to analyze the efficiency of cinnamon compounds as potential drug candidates against three virulent proteins of *H*. *pylori*. These proteins included *virB4*, *virB8* and *virB9*. The predicted 3D structures of these proteins were docked with all the cinnamon compounds. In the virtual screening analysis, *cinnamyl acetate* and *benzyl benzoate* were found to be the potential inhibitors during their respective molecular docking analyses. *Cinnamyl acetate* exhibited significantly good docking interaction with *virB4* and *virB9* proteins while *benzyl benzoate* displayed substantial interaction with *virB8* protein. Afterwards, the molecular dynamics simulations were executed in order to reveal the normal mode analysis (NMA) of the internal coordinates and structural stability of the docked complexes. Our findings suggested that *cinnamyl acetate*-*virB4* and *cinnamyl acetate*-*virB9* complexes showed relatively better structural stability than the *benzyl benzoate*-*virB8* docked complex. In the end, the ADMET analysis was done to evaluate the pharmacokinetic properties of these compounds. Both of the compounds showed the property of insaturation, indicating that they would be chemically more reactive. Both of the compounds also found to possess remarkable drug absorption properties including water solubility and GI absorption. The PBB and VD scores in context of the drug distribution analysis were also up-to the mark. While, in case of drug metabolism assessment, *cinnmayl acetate* did not show inhibition against any of the cytochrome enzymes while *benzyl benzoate* displayed inhibition against CYP1A2 and CYP2C19. However the drug excretion and toxicity properties were satisfactorily found to be followed by both of the compounds. Conclusively, it was found that both of these cinnamon compounds showed most of the required drug-likeness characteristics. This research work can enable the directions towards the real-time formulation of the drugs under study, by following the specified pharmacological protocols. Afterwards, their clinical therapeutic efficacy can also be evaluated. Moreover, this research work can be implied to study other plant-based drugs against certain microbial infections as a reference model.

## Supporting information

S1 File (DOCX)
