# Supplementary material for: Assessing the efficacy of cinnamon compounds against H. pylori through molecular docking, MD Simulations and ADMET analyses
Source: PLoS One. 2024 Mar 11;19(3):e0299378. doi: 10.1371/journal.pone.0299378 (PMC10927141; doi:10.1371/journal.pone.0299378)
Supplement: S1 File — (DOCX) [file pone.0299378.s001.docx]

**1- Amino acid sequences:**

**>ACJ07620.1 VirB8 type IV secretion protein [Helicobacter pylori]**

MSKRSEVLEQFHGGLKNLELQTKRRMDLWGDLKENEEQTLLLGEIENELKQLENKENLKADNNTEFKEEN

QDTKENQPNDLFSLPLPTQTTINGIKEFVEEPMMETEEKETPQNEPIQEKKERIFKNFFSRIGFDKSIAP

TMLFEEVRDASVIYHLEKKLGDYIFYVACFFFGTTALLIILLIVLLPLKQKVPYLVQFSNNKENFALVQK

ADSTITANKALIRSLVGAYVLNRESITHIEQHEKMRQNTIKEQSSNEVWYEFEKLIAHYDSIYTNPLLTR

KVKIANIYLDKDLAYIDIEVSLYHSGELESLKRYKVVMSFEFKKQEINFDSMSLNPTGFIVTGYDVTEIA

ILKDLDEKNKVKDDGVKSRIIHTEKKDPHMSQYKDVKEQ

**>ACF17785.1 VirB9 [Helicobacter pylori]**

MTRVFGGISFAISLLAPLLAENNIQNYNEPMDYSKGDVSLQPFNNQTNQETQPSANHNTQPNSMQTPMPN

QNSYSQPTTPIQNNQPNNAQTQTMLNNAKQFTNNALNNTKQTITNTTNYVNNKLSPKPNDVPISAFKDNR

DLNNQIEPNTSNHETKESDENSDSTLKPMTNFNAIQNNFFAKNRSIKDNAHFIHYTIGDTFNIRLRYAMT

TTFIFDEPIAQVVLGDDIGFSTKMLGEDKEHLISNILLIKPLQIGVDSNLTIIGKSGKVYSFYVFSTTYT

SSKNPALMVYVSSKEYFKHFTSSKKETNKEIKTTHSTPNKDIKVSHAPKKIAKKSPSIKEQNLKSKNPTT

TKTAIENAPLSLNRFKSPSYPPSNALANSRKASENFKVISNSAKTFKEHRHSQDEMKYVDYSAKIKDDGK

FIRIGDNVNHIYINKKKMEYGYVIIDKKKRKWYCLWMCKKVVKSKYKDDLPTQIFNDEQFTYFKFNRSNA

RSKFPVVYKVVDGYDNPVNSRVVGDYLIAEDVSNQWNLKLGKAYLCIEKIAKRAK

**>AIA98891.1 VirB4 type IV secretion protein [Helicobacter pylori]**

MFLGLEGFKRLIFNFLNFCIPSQYSLVQENNINGKFDEKFVLTHKGNLVGAISLSGITYSNFDKEEVATQ

FLYRTQALNELIDGVTLRIVAKRRDIKTQTIYNKDSVSKYAKKIVNAYEKNKEFYITTYYLIIETKSVNI

KGLLEKWKAKMTTDKYKNNDEEEPLKDNPKNSYKVKKNAIENKELQDKATLLAEILNKMSSILTDFEPKF

LSSDDLLNLYAEYCNGHYCDFKYKQGRLSDGNIQSHLYFKKDHFIHDFNGIETFKRFIAVKAYDVDNITS

LALSNLLCEKFSLDIFLTIETMDKERALFFIRERKKRSKNISYQNIDDLEQMVSTDRAQIQKVSLSIMVF

ANSKKELDEKSIIVYNTLKKEGFSAVLESINMRPIFFSFFPERNFLNSRLRPQTSQNIASLIMFEKYQEG

FKENSWGDCPVSVFKNQNGSAHFFNFQAKQGKDKNDNVVGHTMIIGSTGSGKSTFISFLIANLLTKYDMS

VVALDRMNGLEIMTDFFEGQYNTANTDGGFYINPFSLKDTEENRQFLANWIKFMLNIDSDNQQDNKASQS

IDKVIRDTYNYMGDQKNQINLLEIAKNLGSSEQDFNEILKSQGEKIYFKNFQDCLDFSKSPLSVINMDAF

ANDKKLMGLIAMYLFHKLFFEAKEHNKPFFLFIDETKDYIMHPIMFAYITNALAQARKINGTLCMAFQKI

SQVKELGIDKAKSLIGNLSQVIIYPTKDTDELIECGVPLSDSEINFLHNTDMRARQVLVKNIVTNASAFI

EIDLKKDLQELLYILDSNAGNRKILNDLKKTNQETYKEEYLKTKIKKESEKVQYV

**2- SWISS-MODEL Results:**


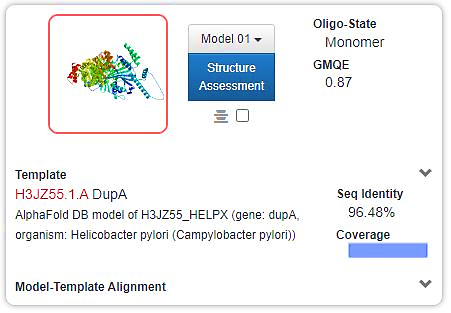


**(a)**

**
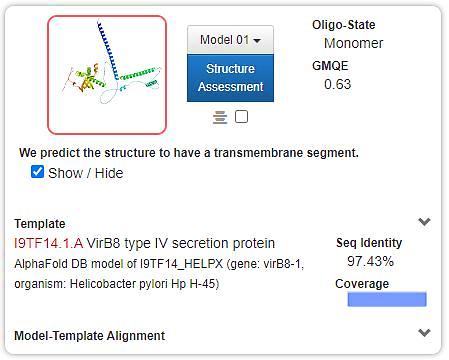
**

**(b)**

**
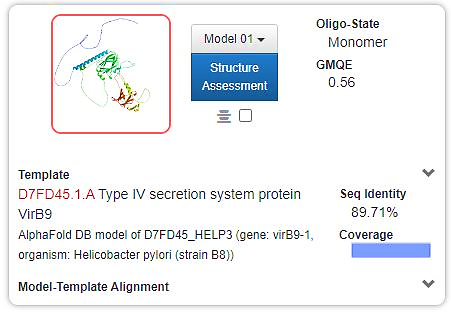
**

**(c)**

**Figure 1.** SWISS-MODEL Results regarding homology modelling of predicted structures; **a:** *virB4 protein,* ***b:*** *virB8 protein,* ***c:*** *virB9 protein*

**3- Ligands retrieved from CHEBI database (**[**https://www.ebi.ac.uk/chebi/**](https://www.ebi.ac.uk/chebi/)**):**

**Table 1.** Cinnamon compounds along their CHEBI ID’s, names and molecular structures

| **CHEBI ID** | **Cinnamon Extracts** | **Structures** |
| --- | --- | --- |
| **CHEBI: 16731** | Cinnamaldehyde | 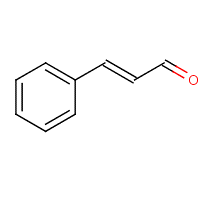 |
| CHEBI: 4917 | Eugenol | 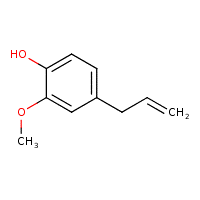 |
| CHEBI: 27386 | Cinnamic acid | 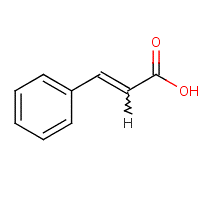 |
| CHEBI: 10357 | Caryophyllene | 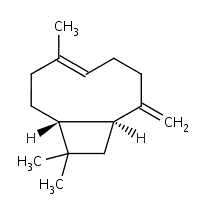 |
| CHEBI: 41237 | Benzyl benzoate | 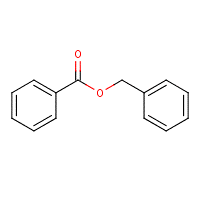 |
| CHEBI: 17580 | Linalool | 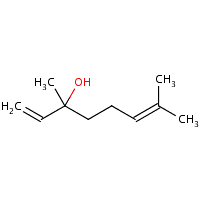 |
| CHEBI: 31402 | Cinnamyl acetate | 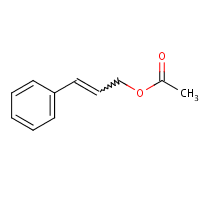 |

- All of the above data were employed to produce the results mentioned in the manuscript file. While, the major findings of this research work are completely available in the manuscript.
